# Association of *TNFRSF10D* DNA-Methylation with the Survival of Melanoma Patients

**DOI:** 10.3390/ijms150711984

**Published:** 2014-07-07

**Authors:** Gudrun Ratzinger, Simone Mitteregger, Barbara Wolf, Regina Berger, Bernhard Zelger, Georg Weinlich, Peter Fritsch, Georg Goebel, Heidelinde Fiegl

**Affiliations:** 1 Department of Dermatology and Venereology, Innsbruck Medical University, Innsbruck 6020, Austria; E-Mails: Gudrun.Ratzinger@i-med.ac.at (G.R.); office@dermapraxis-brixlegg.at (S.M.); Bernhard.Zelger@i-med.ac.at (B.Z.); Georg.Weinlich@i-med.ac.at (G.W.); Peter.Fritsch@i-med.ac.at (P.F.); 2 Department of Obstetrics and Gynecology, Innsbruck Medical University, Innsbruck 6020, Austria; E-Mails: Barbara.Wolf@student.i-med.ac.at (B.W.); Regina.Berger@i-med.ac.at (R.B.); 3 Department of Medical Statistics, Informatics and Health Economics, Innsbruck Medical University, Innsbruck 6020, Austria; 4 Oncotyrol, Center for Personalized Cancer Medicine, Innsbruck 6020, Austria

**Keywords:** cancer biomarker, epigenomics, DNA-methylation, prognosis, translational cancer research

## Abstract

In this retrospective pilot study, the DNA-methylation status of genes that have been demonstrated to be involved in melanoma carcinogenesis was analyzed in order to identify novel biomarkers for the risk assessment of melanoma patients. We analyzed DNA extracted from punch-biopsies from 68 formalin-fixed paraffin-embedded (FFPE) melanoma specimens. Using MethyLight PCR, we examined 20 genes in specimens from a training set comprising 36 melanoma patients. Selected candidate genes were validated in a test set using FFPE tissue samples from 32 melanoma patients. First, we identified the *TNFRSF10D* DNA-methylation status (*TNFRSF10D* methylated *vs.* unmethylated) as a prognostic marker for overall (*p* = 0.001) and for relapse-free survival (*p* = 0.008) in the training set. This finding was confirmed in the independent test set (*n* = 32; overall survival *p* = 0.041; relapse-free survival *p* = 0.012). In a multivariate Cox-regression analysis including all patients, the *TNFRSF10D* DNA-methylation status remained as the most significant prognostic parameter for overall and relapse-free survival (relative-risk (RR) of death, 4.6 (95% CI: 2.0–11.0; *p* < 0.001), RR of relapse, 7.2 (95% CI: 2.8–18.3; *p* < 0.001)). In this study, we demonstrate that *TNFRSF10D* DNA-methylation analysis of a small tissue-punch from archival FFPE melanoma tissue is a promising approach to provide prognostic information in patients with melanoma.

## 1. Introduction

The incidence of melanoma is increasing in white populations worldwide [1]. In advanced disease, median survival is very poor. Melanomas account for 90% of the deaths associated with cutaneous neoplasms [1]. The five-year risk of death of patients with advanced (Stage IV) disease is about 80% [2]. Thus, the recognition of patients at risk for progression is crucial. The most relevant prognostic factors for primary melanoma without metastases are vertical tumor thickness (Breslow’s depth) and the presence or absence of histological ulceration; to a lesser extent, also mitotic activity and invasion level (Clark’s level). However, clinical experience demonstrates that some patients with thin neoplasms face recurrence, metastases and death after surgical excision, while others with thick melanomas do not. New prognostic markers, such as metallothionines or genetic subtypes defined by gene expression profiling, have been established; however, additional reliable markers to select patients for early therapy are still lacking [3,4]. Besides genetic alterations, changes in the status of DNA-methylation, a type of epigenetic alteration, are among the most common molecular alterations in human neoplasia, including melanoma [5,6,7,8,9]. Irreversible silencing of certain genes by DNA-methylation might enable cells to acquire new capabilities that may drive tumorigenesis. Here, we analyzed the DNA-methylation status of the 5' regions of 20 different genes that are involved in melanoma carcinogenesis and that have previously been shown to be aberrantly methylated in melanoma [7,9,10,11,12,13,14,15,16,17] or other human cancers [18].

In the present pilot study, we aimed to explore whether differences in the DNA-methylation pattern from formalin-fixed paraffin-embedded (FFPE) tissues may be useful as a prognostic marker for the further outcome of non-metastatic melanoma patients.

## 2. Results and Discussion

### 2.1. Training Set for Selection of Relevant Genes

Using MethyLight PCR, we analyzed 20 genes that have been demonstrated to be involved in melanoma tumorigenesis (*APC*, *CDH13*, *CDKN2A*, *CYP1B1*, *ENC1*, *ESR1*, *LOX*, *MAGEA1*, *MIR34A*, *PPP1R3C*, *PYCARD*, *RARB*, *RARRES1*, *RASSF1*, *SFN*, *SOCS1*, *TIMP3*, *TNFRSF10C*, *TNFRSF10D* and *TP73*) [7,9,10,11,12,13,14,15,16,17,18] from FFPE tissue punches of 36 patients (training set). Association analysis between clinicopathological features, sex, age and methylation status of the analyzed genes revealed no significant differences. Only *MAGE1A* showed higher DNA-methylation values (the percentage of fully methylated reference, PMR) in women (*p* = 0.001). In this set, we identified age, the Clark level, ulceration, tumor thickness, mitotic rate and *TNFRSF10D* DNA-methylation status (*TNFRSF10D* methylated *vs.* unmethylated) as univariate prognostic markers for overall survival (*p* = 0.049, 0.001, 0.015, 0.008, 0.026 and 0.001). The Clark level, ulceration, tumor thickness and *TNFRSF10D* methylation status were found as univariate prognostic markers for relapse-free survival (*p* < 0.001, 0.046, 0.008, 0.008; Table 1A).

### 2.2. Test Set for the Validation of Relevant Genes

For validation of the results obtained with the training set, we analyzed the *TNFRSF10D* DNA-methylation status in an independent test set consisting of FFPE tissues of 32 melanoma patients. Furthermore, in this analysis, *TNFRSF10D* DNA-methylation was confirmatively shown to be highly significantly associated with a poor overall and relapse-free survival (*p* = 0.041 and *p* = 0.012, respectively; Table 1B).

**Table 1:** Univariate survival analysis in melanoma patients. (**A**) Training set; (**B**) Test set; (**C**) Overall samples. Significant *p*-values in bold.

(A) Training Set				
Variable	Overall Survival		Relapse-Free Survival	
	No. Patients (Died/Total)	*p*-Value (Log-Rank-Test)	No. Patients (Relapsed/Total)	*p-*Value (Log-Rank-Test)
**Sex**				
Male	12/24	0.712	11/24	0.717
Female	5/12		5/12	
**Age**				
<60	9/24	**0.049**	10/24	0.239
≥60	8/12		6/12	
**Clark level**				
2/3	4/11	**0.001**	3/11	**<0.001**
4	9/21		9/21	
5	4/4		4/4	
**Ulceration**				
No	7/22	**0.015**	7/22	**0.046**
Yes	10/14		9/14	
**Tumor thickness**				
≤2 mm (T1/T2)	5/13	**0.008**	6/13	**0.008**
2.01–4 mm (T3)	2/8		1/8	
≥4.01 mm (T4)	10/15		9/15	
**Mitotic rate**				
≤1/mm^2^	3/12	**0.026**	3/12	0.052
>1/mm^2^	14/24		13/24	
**Interferon alpha therapy**				
No	15/28	0.302	14/28	0.374
Yes	2/8		2/8	
***TNFRSF10D***				
Unmethylated	11/29	**0.001**	11/29	**0.008**
Methylated	6/7		5/7	
**(B) Test Set**				
**Sex**				
Male	5/12	0.929	5/12	0.98
Female	10/20		9/20	
**Age**				
<60	7/22	**0.003**	7/22	**0.031**
≥60	8/10		7/10	
**Clark level**				
2/3	3/7	**<0.001**	3/7	**<0.001**
4	6/18		5/18	
5	6/6		6/6	
**Ulceration**				
No	11/23	0.887	10/23	0.942
Yes	4/9		4/9	
**Tumor thickness**				
≤2 mm (T1/T2)	5/12	0.919	5/12	0.992
2.01–4 mm (T3)	7/13		6/13	
≥4.01 mm (T4)	2/5		2/5	
**Mitotic rate**				
≤1/mm^2^	6/18	0.077	6/18	0.141
>1/mm^2^	9/14		8/14	
**Interferon alpha therapy**				
No	9/24	**0.003**	8/24	**0.002**
Yes	6/8		6/8	
***TNFRSF10D***				
Unmethylated	11/28	**0.041**	10/28	**0.012**
Methylated	4/4		4/4	
**Sex**				
Male	17/36	0.683	16/36	0.734
Female	15/32		14/32	
**Age**				
<60	16/46	**<0.001**	13/22	**0.013**
≥60	16/22		23/74	
**Clark level**				
2/3	7/18	**<0.001**	6/18	**0.011**
4	15/39		14/39	
5	10/10		10/10	
**Ulceration**				
No	18/45	0.085	17/45	0.116
Yes	14/23		13/23	
**Tumor thickness**				
≤2 mm (T1/T2)	10/25	0.084	11/25	0.113
2.01–4 mm (T3)	9/21		7/21	
≥4.01 mm (T4)	12/20		11/20	
**Mitotic rate**				
≤1/mm^2^	9/30	**0.005**	9/30	**0.012**
>1/mm^2^	23/38		21/38	
**Interferon alpha therapy**				
No	24/52	0.261	22/52	0.24
Yes	8/16		8/16	
***TNFRSF10D***				
Unmethylated	22/57	**<0.001**	21/57	**<0.001**
Methylated	10/11		9/11	

### 2.3. Overall Prognostic Significance Merging the Training and the Test Set

**Figure 1:**
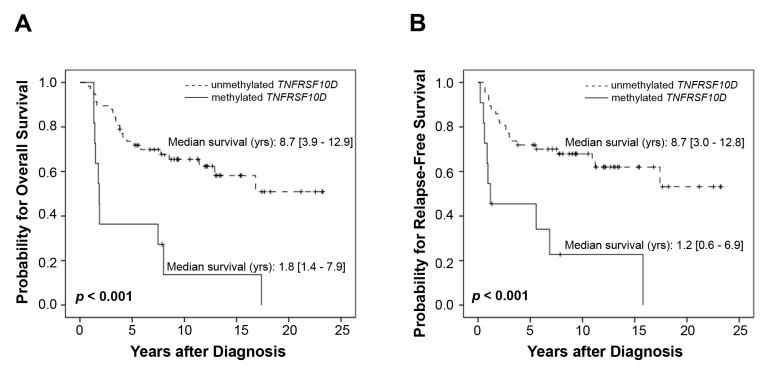
Kaplan–Meier survival analysis: (**A**) the overall survival and (**B**) relapse-free survival of 68 melanoma patients.

The comprehensive univariate analysis of all 68 patients together identified age (<60 *vs.* ≥60), the Clark level, mitotic rate and *TNFRSF10D* DNA-methylation status as prognostic parameters for poor overall survival (*p* < 0.001, <0.001, 0.005, <0.001; Table 1C), as well as for poor relapse-free survival (*p* = 0.013, 0.011, 0.012, <0.001; Table 1C). The Kaplan–Meier survival analysis for *TNFRSF10D* DNA-methylation is depicted in Figure 1.

Finally, we analyzed all patients, including the variables, sex, age, Clark level, presence of ulceration, tumor thickness, interferon alpha therapy and *TNFRSF10D* DNA-methylation status, using a multivariate Cox regression model. The *TNFRSF10D* DNA-methylation status remained the most significant prognostic parameter for overall and relapse-free survival. Patients with methylated *TNFRSF10D* showed a 4.6-fold higher risk of death (95% CI: 2.0–11.0; *p* < 0.001) and a 7.2-fold higher risk of relapse (95% CI: 2.8–18.3; *p* < 0.001) than patients with unmethylated *TNFRSF10D* (Table 2). After exclusion of a subgroup of 16 patients, who received an adjuvant interferon alpha therapy, *TNFRSF10D* DNA-methylation still remained the highest significant prognostic parameter in the multivariate analysis for overall (*p* = 0.004) and relapse-free survival (*p* = 0.001).

**Table 2:** Multivariate survival analysis of 68 melanoma patients. RR, relative-risk. Significant *p*-values in bold.

Variable	Overall Survival		Relapse-Free Survival	
	RR of Death (95% CI)	*p*-Value	RR of Relapse or Progression (95% CI)	*p*-Value
**Sex**				
Male	0.5 (0.2–1.6)	0.11	0.4 (0.2–0.9)	**0.03**
Female				
**Age**				
<60	2.1 (0.9–4.6)	0.07	1.6 (0.75–4.1)	0.20
≥60				
**Clark level**				
2/3	3.0 (1.6–5.9)	**0.001**	4.9 (2.4–10.1)	**<0.001**
4				
5				
**Ulceration**				
No	1.4 (0.6–3.5)	0.47	1.1 (0.5–2.8)	0.78
Yes				
**Tumor thickness**				
≤2 mm (T1/T2)	0.9 (0.6–1.6)	0.82	0.8 (0.5–1.3)	0.39
2.01–4 mm (T3)				
≥4.01 mm (T4)				
**Mitotic rate**				
≤1/mm^2^	1.4 (0.5–3.9)	0.49	1.6 (0.6–4.5)	0.37
>1/mm^2^				
**Interferon alpha therapy**				
No	2.2 (0.8–5.9)	0.11	2.7 (1.1–7.5)	**0.03**
Yes				
***TNFRSF10D***				
Unmethylated	4.6 (2.0–11.0)	**<0.001**	7.2 (2.8–18.3)	**<0.001**
Methylated				

### 2.4. Discussion

The prognostic biomarkers currently used in melanoma do not adequately predict disease recurrence and overall survival in a significant subset of patients. Therefore, novel biomarkers are highly required to overcome these problems.

In this pilot study, we identified *TNFRSF10D* DNA methylation status in paraffin-embedded melanoma tissues to be an independent prognostic biomarker for relapse-free survival and overall mortality in non-metastatic melanoma patients. Surprisingly, ulceration and tumor thickness were significantly associated with survival only in the training set, whereas the invasion level (Clark’s level) was a significant, prognostic feature consistently in all analyses performed in this work. Due to the small sample size of this pilot study, a subsequent validation study in a larger, independent patient cohort must be performed. In our study, the prognostic value of *TNFRSF10D* concerns mainly the group of patients with methylated *TNFRSF10D* in the tumors. Recently, *TNFRSF10D* DNA-methylation has also been shown to be an independent prognostic and predictive marker in the serum of patients with *MYCN* nonamplified neuroblastoma [19]. Interestingly, recently published data indicated that *TNFRSF10D* is epigenetically silenced in human melanoma [9,16], as well as in cancers of breast, lung, mesothelioma, prostate, bladder, cervix, ovary, brain and in hematopoietic malignancies [20]. Bonazzi *et al.* found that 72% of the analyzed melanoma cell lines had no *TNFRSF10D* mRNA expression and that the transcript level of *TNFRSF10D* was correlated inversely with promoter methylation [16]. They identified that 66% of the analyzed cell lines and 30% of the analyzed fresh frozen melanoma samples showed a high degree of methylation. In our study, we identified *TNFRSF10D* DNA-methylation in only 16% of all analyzed specimens. This discrepancy probably reflects the different levels of *TNFRSF10D* DNA-methylation in melanoma cell lines, metastatic tumor tissues and primary tissues. Bonazzi *et al.* observed a five-fold average increase in *TNFRSF10D* mRNA expression in five melanoma cell lines after treatment with the DNA-demethylating agent, 5-aza-2'-deoxycytidine [16]. *TNFRSF10D* belongs to the tumor necrosis factor (TNF) receptor superfamily. This receptor contains an extracellular tumor necrosis factor-related apoptosis-inducing ligand (TRAIL) binding domain, a transmembrane domain and a truncated cytoplasmic death domain. The second known TRAIL decoy receptor, TNFRSF10C, lacks this intracellular death domain completely. Therefore, both receptors appear unable to induce apoptosis. Considering that *TNFRSF10D*, as well as *TNFRSF10C* have been presumed to function as oncogenes, because of their postulated anti-apoptotic effect [20], our data seem to be controversial at first sight. However, recently, Venza *et al.* have shown that an ectopic overexpression of *TNFRSF10C* and/or *TNFRSF10D* in melanoma cell lines led to a significant reduced growth rate and a clear increased apoptotic response [21]. In the context of our data, it seems that the methylation and silencing of *TNFRSF10D* may represent a special feature of more aggressive cancer cells.

## 3. Experimental Section

### 3.1. The Patient Study Cohort and Study Design

We retrospectively analyzed prospectively collected melanoma specimens (FFPE tissues) from melanoma patients treated at the Department of Dermatology and Venereology, Innsbruck Medical University, Innsbruck, Austria. Samples have been collected during primary surgery. Sixty-eight patients (32 women and 36 men) diagnosed between 1983 and 2004 with primary, invasive, non-metastasized melanoma were included in this study (T1–T4, tumor-node-metastasis (TNM) classification American Joint Committee of Cancer 2001). Patients had no metastases at the time of diagnosis or surgery, respectively. Thirty-four melanomas were located on the trunk, 34 on the limbs. The tumor thickness was 0.5–2 mm (T1/T2), 2.01–4 mm (T3) and >4 mm (T4) in 25, 23 and 18 patients, respectively; tumor thickness was unknown in two patients. Twenty-three patients showed ulcerated melanomas. All patients underwent surgery with 1–2 cm excision margins, according to standard guidelines, and 16 patients received adjuvant interferon alpha. The median age was 53.4 years (21.9–90.7 years). Thirty and 32 patients relapsed/died, respectively, due to the consequences of the melanoma after a median follow up period of 2.0 (interquartile (IQ)-range 6.2) and 3.4 (IQ-range 5.5) years, respectively. The patient study cohort was *a priori* randomly split into a training and a test set, consisting of 36 and 32 patients, respectively. The study was approved by the local medical research ethics committee (Reference Number UN3856, 26 January 2010) and conducted in accordance with the Declaration of Helsinki. Reporting Recommendations for Tumor Marker Prognostic Studies (REMARK) were adhered to where applicable [22,23].

### 3.2. DNA Extraction and Bisulfite Conversion from Formalin-Fixed Paraffin-Embedded (FFPE) Tissues

DNA was isolated from punches gained from FFPE melanoma specimens using the DNeasy Tissue Kit (Qiagen, Hilden, Germany) in order to assure that mainly melanoma tissue was collected.

Sodium bisulfite-modification of genomic DNA (700 ng) was performed using the EZ DNA Methylation-Gold Kit (Zymo Research, Orange, CA, USA), according to the manufacturer’s instructions.

### 3.3. DNA Methylation Analysis

Thirty nanograms of bisulfite-modified DNA were analyzed by means of MethyLight analysis, as described previously [24]. Briefly, two sets of primers and probes, designed specifically for bisulfite-converted DNA, were used: a set representing fully methylated DNA for the gene of interest and a reference set, collagen, type II, alpha 1 (*COL2A1*), to normalize for input DNA. Primers and probes for *APC*, *COL2A1*, *CDH13*, *CDKN2A*, *CYP1B1*, *ENC1*, *ESR1*, *LOX*, *MAGEA1*, *MIR34A*, *PYCARD*, *PPP1R3C*, *RARB*, *RARRES1*, *RASSF1*, *SFN*, *SOCS1*, *TIMP3*, *TNFRSF10C*, *TNFRSF10D* and *TP73* are listed in Tables S1 [25,26].

To control for the amount of input bisulfite-modified DNA, this value was normalized to the extent of amplification of a *COL2A1* DNA sequence lacking CpG dinucleotides. The specificity of the reactions for methylated DNA was confirmed separately using SssI (New England Biolabs, Ipswich, MA, USA)-treated human white blood cell DNA (heavily methylated). The SssI treated DNA was additionally used for the standard curve preparation, which is required for the quantification. The percentage of fully methylated molecules at a specific locus was calculated by dividing the GENE:COL2A1 ratio of a sample by the GENE:COL2A1 ratio of SssI-treated white blood cell DNA and multiplying by 100 (the percentage of fully-methylated reference, PMR). PMR values have been calculated for all analyzed genes. If less than 50% of the samples were methylated for a specific gene (a gene was deemed methylated if the PMR value was >0), we categorized the samples in “unmethylated” and “methylated” and performed the analyses with these dichotomized variables. Only in 3 genes were more than 50% of the samples methylated (*ESR1*, *SFN* and *MAGE1*). For these genes we used the PMR values for the following statistical analysis.

### 3.4. Mitotic Rate

The mitotic rate per square millimeter of tumor tissue was evaluated by counting mitotic figures on hematoxylin and eosin (H&E)-stained tissue sections.

### 3.5. Statistical Analysis

Disease-free and overall survival were calculated from the date of diagnosis to the date of relapse, death or last follow-up. Disease-free and overall survival curves were calculated with the Kaplan–Meier method. Univariate analysis of overall survival according to clinicopathological factors (age, Clark-level, tumor thickness, presence of ulcerations, *etc.*) or DNA-methylation status was performed using a two-sided log rank test. A multivariate Cox proportional hazards model was applied to estimate the prognostic effect of *TNFRSF10D* DNA-methylation. A *p-*value <0.05 was considered statistically significant. SPSS 19.0 was used for all statistical analyses (SPSS Inc., Chicago, IL, USA).

## 4. Conclusions

Our data demonstrate that DNA-methylation analysis of the *TNFRSF10D* promoter from a small tissue punch from archival paraffin-embedded melanoma tissue is able to provide independent prognostic information in order to identify patients with a higher risk for aggressive progress.

Further studies are needed to elucidate how *TNFRSF10D* promoter hypermethylation is associated with poor prognosis and aggressive proliferation in melanoma. Additionally, further research needs to be conducted to assess if *TNFRSF10D* hypermethylation in serum samples from melanoma patients could be an indicator of poor prognosis in melanoma, as has been shown in neuroblastoma patients [19].

## Supplementary Files

Supplementary File 1
