# Supplementary material for: Association of TNFRSF10D DNA-Methylation with the Survival of Melanoma Patients
Source: Int J Mol Sci. 2014 Jul 7;15(7):11984–95. doi: 10.3390/ijms150711984 (PMC4139825; doi:10.3390/ijms150711984)
Supplement: Supplementary File 1 [file ijms-15-11984-s001.pdf]

Supplementary Information

Table S1. MethyLight Primer information.

Gene	Forward Primer Sequence (5'–3')	Reverse Primer Sequence (5'–3')	TaqMan Probe Sequence (5'-FAM; 3'-BHQ1)	References or Position Relative to Transcription Start Site
APC	GAACCAAAACGCTCCCCAT	TTATATGTCGGTTACGTGCGTTTATAT	CCCGTCGAAAACCCGCCGATTA	[25]
CDH13	AATTTCGTTCGTTTTGTGCGT	CTACCCGTACCGAACGATCC	AACGCAAAACGCGCCCGACA	[25]
CDKN2A	TGGAGTTTTCGGTTGATTGGTT	AACAACGCCCGCACCTCCT	ACCCGACCCCGAACCGCG	[25]
COL2A1	TCTAACAATTATAAACTCCAACCACCAA	GGGAAGATGGGATAGAAGGGAATAT	CCTTCATTCTAACCCAATACCTATCCCACCTCTAAA	[26]
CYP1B1	GTGCGTTTGGACGGGAGTT	AACGCGACCTAACAAAACGAA	CGCCGCACACCAAACCGCTT	[25]
ENC1	TCTACCCTAAACCAAACGACTACAAA	GGCGATTGGTTGAAAAGTACGT	CAACCGAAACGCAATCCTACGAAACG	−79 to +2
ESR1	GGCGTTCGTTTTGGGATTG	GCCGACACGCGAACTCTAA,	CGATAAAACCGAACGACCCGACGA	[25]
LOX	CCTTTCCCCTTTCTCAATCCTAAA	CGGTCGGGTTAGAACGGTT	AAACGACGCGACAATCCCGAAAAAC	+24 to +170
MAGEA1	CTCGCTTCTCTCTACCCCACAA	GTAGGATTCGGTTTTCGTTAGGAAA	CGCAAATCAATAACGTCACATCCGAACA	−72 to +12
MIR34A	TCCTTCCTACTCGTACCACCAAA	AGGTGGAGGAGATGTCGTTGTT	CGTCTCTCCAACCCGAAATCCGAAAAA	−192 to −82
PPP1R3C	GCCCCGCCTAAACGAAAC	ATTAGTCGCGTAACGGGAATTTT	CTCTCTCTTAACCGACGCCCGCTACA	−288 to −183
PYCARD	TTGGAGATTTACGGCGTCG	ACCCTAATACGTAACCGCCTACAA	CATCTCCTACAAACCCATATCGCGCAA	[25]
RARB	TTTATGCGAGTTGTTTGAGGATTG	CGAATCCTACCCCGACGATAC	CTCGAATCGCTCGCGTTCTCGACAT	[25]
RARRES1	GGCGAGTCGGATCGGAA	CGCAAACTCCTACAACAAACGA	CGCGCGACGCTTCACTTCTTCAA	[25]
RASSF1	ATTGAGTTGCGGGAGTTGGT	ACACGCTCCAACCGAATACG	CCCTTCCCAACGCGCCCA	[25]
SFN	GAGGAGGGTTCGGAGGAGAA	ATCGCACACGCCCTAAAACT	TCTCCCGATACTCACGCACCTCGAA	[25]
SOCS1	GCGTCGAGTTCGTGGGTATTT	CCGAAACCATCTTCACGCTAA	ACAATTCCGCTAACGACTATCGCGCA	[25]
TIMP3	GCGTCGGAGGTTAAGGTTGTT	CTCTCCAAAATTACCGTACGCG	AACTCGCTCGCCCGCCGAA	[25]
TNFRSF10C	GGGAAGAGCGTATTTGGCG	TCCCCTAACTCCGACGACG	CGAACATACCCGACCGCAAATAACCA	[25]
TNFRSF10D	GGGAAGAGCGTATTTGGCG	TCCCCTAACTCCGACGACG	TACCCGACCGCAAACGACCCG	[25]
TP73	GGGTCGGGTAGTTCGTTTTG	CGATTTCGCTACGTCCCCT	AACCTCCGAACGAATACGCGAACGAA	[25]
